# MicroRNAs in Hepatocellular Carcinoma: Regulation, Function, and Clinical Implications

**DOI:** 10.1155/2013/924206

**Published:** 2013-02-04

**Authors:** Jie Sun, Haiqi Lu, Xian Wang, Hongchuan Jin

**Affiliations:** Laboratory of Cancer Epigenetics, Department of Medical Oncology, Biomedical Research Center, Sir Run Run Shaw Hospital, School of Medicine, Zhejiang University, Hangzhou, Zhejiang 310016, China

## Abstract

Hepatocellular carcinoma (HCC) is one of the most common cancers worldwide and the third cause of cancer-related death. Poor understanding of the mechanisms underlying the pathogenesis of HCC makes it difficult to be diagnosed and treated at early stage. MicroRNAs (miRNAs), a class of noncoding single-stranded RNAs of ~22 nucleotides in length, posttranscriptionally regulate gene expression by base pairing with the 3′ untranslated regions (3′UTRs) of target messenger RNAs (mRNAs). Aberrant expression of miRNAs is found in many if not all cancers, and many deregulated miRNAs have been proved to play crucial roles in the initiation and progression of cancers by regulating the expression of various oncogenes or tumor suppressor genes. In this Paper, we will summarize the regulations and functions of miRNAs aberrantly expressed in HCC and discuss the potential application of miRNAs as diagnostic and prognostic biomarkers of HCC and their potential roles in the intervention of HCC.

## 1. Introduction

Being one of the most frequently diagnosed cancer worldwide, liver cancer is the second leading cause of cancer-death in men and the sixth leading cause of cancer-related death in women [1]. Hepatocellular carcinoma (HCC), which account for 70% to 85% of the primary liver cancer cases, is rarely detected at its early stage, resulting in a short survival of few months [2]. About 90% of HCC cases arise from cirrhosis, which can be attributed to a wide range of factors including chronic viral hepatitis B or C (HBV or HCV) infections, alcohol abuse, nonalcoholic steatohepatitis (NASH), autoimmune hepatitis, primary biliary cirrhosis (PBC), and carcinogens exposure [3]. Considerable progresses on unraveling molecular mechanisms of HCC have been achieved recently, paving the way to the early detection and treatment of HCC.

MicroRNAs (miRNAs), a class of noncoding RNAs of ~22 nucleotides in length found both in plants and animals, have emerged as key posttranscriptional regulators of gene expression. It has been reported that many miRNAs are involved in human cancers, such as lung, breast, brain, liver, colorectal cancer, and leukemia. By targeting different genes in tumor development, miRNAs function as oncogenes or tumor suppressor genes. In this paper, we will summarize the process and the regulation of miRNA biogenesis, as well as our current knowledge about the biological relevance of miRNAs to HCC. Then, we will discuss the potential application of miRNAs as predictive, diagnostic, and prognostic biomarkers of HCC and their potential roles in cancer treatment. 

Since the first discovered miRNA lin-4 by Victor Ambros and his colleagues in *Caenorhabditis elegans,* more than 20,000 miRNAs have been identified in 193 species (Sanger miRBase release 19; http://www.mirbase.org/) [4]. miRNAs downregulate the expression of specific genes predominantly by base pairing with the 3′ untranslated regions (3′UTRs) of target messenger RNAs (mRNAs), leading to translational inhibition, transcript destabilization, or both [5]. However, recently, findings indicate that miRNAs can also target the 5′UTRs and coding regions of mRNAs [6, 7]. 

## 2. The Biogenesis and Maturation of miRNAs

**Figure 1:**
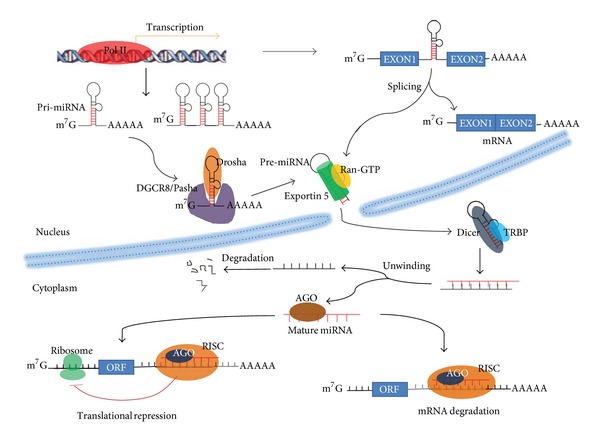
The biogenesis of microRNAs is summarized. Genes encoding microRNAs are transcribed mainly by Pol II (DNA-dependent RNA polymerase II) with the products as the pri-miRNAs that are processed into pre-miRNA by DGCR8/Drosha. Pre-miRNAs are exported into the cytoplasm where they are further converted into the mature forms by the TRBP/Dicer. Mature miRNAs bind to the target mRNAs to inhibit their expression. ORF: open-reading frame; RISC: RNA-induced silencing complex.

In the nucleus, miRNAs genes are transcribed mainly by RNA polymerase II to generate primary miRNA transcripts (pri-miRNAs) that consist of one or more hairpin structures and finally produce one or more functional miRNAs [8]. Like protein-coding mRNAs, pri-miRNAs are usually capped at the 5′ end and polyadenylated at the 3′ end [9]. Pri-miRNAs are then cleaved into ~70 nt hairpin-structured precursors (pre-miRNAs) with a 5′ phosphate and a 3′ 2nt overhang by a multiprotein complex called microprocessor which consists of Drosha, an RNase III enzyme, and DGCR8/Pasha, a double-stranded RNA-binding domain protein (dsRBD) [10]. The 3′ 2nt overhang is recognized by exportin-5 which transports pre-miRNAs to the cytoplasm via an Ran-GTP-dependent mechanism [11]. In the cytoplasm, pre-miRNAs are further processed to ~22 nt duplex by Dicer, a second RNase III endonuclease, and the dsRBD proteins TRBP/PACT [12]. Finally, the two miRNA strands are unwound, and one of the strands associates with an argonaute (AGO) protein within the RNA-induced silencing complex (RISC) where they regulate gene expression by mRNA degradation or translational repression, while another miRNA strand is quickly degraded [13] (Figure 1).

However, some miRNAs are not generated as described earlier. Mirtrons, for example, are produced from spliced introns as debranched introns that mimic the structural features of pre-miRNAs and enter to miRNA-processing pathway without Drosha-mediated cleavage [14]. In addition, some small nucleolar RNAs (snoRNAs) [15], transfer RNAs (tRNAs) [16], and endogenous short hairpin RNAs (shRNAs) [17] can also be processed into miRNA-like molecules in a microprocessor-independent manner.

### 2.1. Transcriptional Regulation of miRNAs

Transcription is an important step for miRNA expression regulation. Many characteristics of protein-coding genes, such as CpG islands, TATA box, TFIIB recognition, initiator elements, and histone modifications, also present in the promoters of miRNA genes [18], suggesting that the transcription regulators of miRNA like transcription factors (TFs), enhancers, and silencing elements may be similar to protein-coding genes. For instance, myogenin and myoblast determination 1 (MyoD1) can bind to the upstream of miR-1 and miR-133 loci and induce their transcription during myogenesis [19]. The proto-oncogene c-Myc which regulates ~10–15% of human genes, binds to E-boxes and activates the transcription of miR-17-92 cluster [20], whereas the tumor suppressor p53 transactivates miR-34, which consequently suppresses the transcriptional activity of *β*-catenin [21].

Furthermore, miRNAs can autoregulate their own transcription by targeting some transcription factors to establish negative or positive feedback loops. For example, miR-133b can regulate the maturation and function of midbrain dopaminergic neurons within a negative feedback circuit that includes the paired-like homeodomain transcription factor PITX3, the regulator and direct target of miR-133b [22]. Zinc-finger E-box-binding protein ZEB1/SIP1 and the miRNA-200 family represent another example of a complex double-negative feedback regulation. miRNAs in miR-200 family play major roles in maintaining the epithelial phenotype by preventing the expression of ZEB1/SIP1, which can bind to the transcription start site of miR-200 family genes and repress their expression in mesenchymal cells [23].

Similar to protein-coding genes, the expression of miRNAs is also regulated by epigenetic mechanisms including DNA methylation and specific histone modifications. MiR-127, the first reported epigenetically regulated miRNA, is upregulated after treatment with demethylation agents 5-aza in several cancer cell lines [24]. Moreover, inhibition of histone deacetylases (HDACs) results in transcriptional changes of ~40% miRNAs genes [25]. For example, Sampath and his colleagues demonstrated that HDACs mediated the silencing of miR-15a, miR-16, and miR-29b in chronic lymphocytic leukemia [26].

### 2.2. Posttranscriptional Regulation of miRNAs

The expression of miRNAs is also controlled by the posttranscriptional maturation. For example, many pri-miRNAs are expressed during early mouse development but are not efficiently processed into mature miRNAs [27]. Moreover, the expression of individual miRNAs in a genomic cluster and processed from the same pri-miRNA is sometimes different at the mature form level [28].

The first step of miRNA processing is catalyzed in the nucleus by Drosha which is associated with DGCR8 and other proteins to form the microprocessor complex. Downregulation of the expression level of either Drosha or DGCR8 by RNAi leads to the reduction of both pre-miRNAs and mature miRNAs [10], and defects in the Drosha processing step contribute to widespread downregulation of miRNAs in primary tumors [27]. Some transcription factors, such as p53, receptor-regulated SMADs (R-Smads), and estrogen receptor *α* (ER*α*), can also participate in the rapid regulation of miRNA expression in response to extracellular stimuli by interacting with the DEAD-box RNA helicases p68 (DDX5) and/or p72 (DDX17). Both of them are components of the Drosha microprocessor complex [29–31]. 

The expression of let-7 miRNA controlled by Lin-28 shows a complicated model of posttranscriptional regulation of miRNA expression. The RNA-binding protein Lin28 selectively represses the let-7 family miRNAs biogenesis by binding to the terminal loop of pre- and pri-let-7 miRNAs [32]. During cell differentiation, the increase of mature let-7 results from the decrease of Lin28 [33]. Interestingly, Lin28 is downregulated by let-7 miRNAs, presenting a double-negative feedback loop in cell differentiation [34].

## 3. Involvement of miRNAs in HCC Development

### 3.1. miRNAs and HCC-Associated Virus Infection

Chronic infections with either HBV or HCV increase the relative risk of liver cancer greatly. These chronic viral infections are present in more than 70% of HCC cases, and iatrogenic interventions against these viruses significantly reduce the risk of HCC development [35]. Cellular miRNAs have shown able to regulate HBV infection at the transcription level either by targeting cellular transcription factors required for HBV gene expression or by a directly binding to HBV transcripts [36]. For instance, miR-155 can downregulate HBV transcription by inhibiting the expression of CCAAT/enhancer-binding protein (C/EBP-*β*), which binds to the Enhancer II, core promoter and S-promoter of HBV cccDNA and activates the transcription of HBV cccDNA [37]. ER*α*-targeting miR-18a is overexpressed in female HCC tissues, thus blocking ER*α*-mediated suppression of HBV transcription [38]. This maybe explain why hepatocellular carcinoma predominantly affects men, with an incidence typically more than twofold higher in males than in females [1].

miRNAs can target important players in DNA methylation and histone modification that play crucial roles in HBV cccDNA transcription. For example, miR-152 and miR-148a target DNA (cytosine-5)-methyltransferase 1 (DNMT-1) can methylate viral DNA and inhibit HBV replication [39]. Similarly, miR-1 regulates the expression of HDAC4 that can inhibit the replication of HBV [40]. On the other hand, HBV-encoded proteins can influence cellular miRNA expression. Wang et al. compared the expression of 286 cellular miRNAs before and after the expression of HBx protein in HepG2 cells [41]. HBx was found to significantly upregulate the expression of 7 miRNAs but downregulate the expression of 11 cellular miRNAs such as the let-7 family miRNAs [42, 43]. miRNAs in the let-7 family are commonly downregulated in various cancers including HCC and target and downregulate a number of proteins playing important roles in tumorigenesis and metastasis, such an Ras [44], high-mobility group AT-hook 2 (HMGA2) [45], myc [46], and signal transducer and activator of transcription 3 (STAT3) [41].

HCV infection is another independent risk factor for HCC. MiR-196 plays a protective role in HCV-induced HCC by upregulating heme oxygenase (decycling) 1 (HMOX1) expression and inhibiting HCV transcription [47]. 

### 3.2. miRNAs and Other Factors Related with HCC Development

Cirrhosis caused by chronic alcohol consumption is another risk factor of HCC especially in western countries. MiR-217 could promote ethanol-induced fat accumulation in hepatocytes by downregulating SIRT1 [48]. In a miRNA profiling study, Ladeiro et al. found that miR-126* was decreased in alcohol-related HCC [49]. Hepatic specimens from mice fed with an ethanol-containing diet indicated a decreased expression of miR-199 and miR-200, which are commonly downregulated in HCC [50].

miRNAs are also involved in the pathogenesis of NASH, an increasingly important risk factor for HCC in recent years. Unsaturated fatty acids have been shown to increase miR-21 expression which downregulates the expression of tumor suppressor phosphatase and tensin homolog (PTEN) [51]. MiR-155 which targets another tumor suppressor C/EBP-*β* [52] is consistently upregulated in choline-deficient and amino acid-defined (CDAA) fed mice [37]. 

Carcinogen exposure induces malignant transformation often accompanied with miRNA deregulation. For instance, after continuously being exposed to a low concentration of microcystin-LR, a hepatocarcinogen, the human hepatic cell line WRL-68 showed aberrant miRAN expression including the up-regulation of oncogenic miRNA, miR-21, and miR-221 [53]. Deregulation of miRNA expression also was found in the liver of mice treated with microcystin-LR and such deregulated miRNAs including miR-125b, miR-34a, and miR-21 which play crucial roles in liver tumorigenesis [54]. Furthermore, miR-191 that was known to regulate a variety of oncogenic pathways was found to be upregulated by dioxin, a known liver carcinogen [55]. These miRNA alterations may be used to develop methods monitoring environmental carcinogens.

### 3.3. miRNAs Deregulated in HCC

Many reports had shown miRNA deregulation in HCCs and a list of aberrantly expressed miRNAs in HCC, has been summarized in Table 1. Like transcription factors, miRNAs play key roles in regulating diverse cellular pathways. Moreover, aberrant miRNA expression can be used as the signature to detect or characterize different type of HCC.

## 4. Biological Relevance of Deregulated miRNAs in HCC

miRNAs have been proved to exert their functions either as oncogenes or tumor suppressor genes in human cancers. Deregulated miRNAs in cancer cells have been found to contribute to most if not all hallmarks of malignant transformation including sustained proliferative signaling, resistant to cell death, immortality, angiogenesis, invasion, and metastasis [56]. 

### 4.1. Regulation of Cell Proliferation and Survival by Deregulated MicroRNAs in HCC

Under normal physiological conditions, cell proliferation and death are finely balanced by many regulators involved in cell cycle, growth, and apoptosis. However, these regulators are targeted by miRNAs deregulated in HCC.

Several miRNAs have been reported to be implicated in cell cycle regulation. For example, miR-26a and miR-195, which were found to be frequently downregulated in HCC, cooperate to overcome the G1/S cell cycle blockade through the repression of E2F expression [57, 58]. In contrast, E2F1-targeting miR-106b and miR-93 promote the pathogenesis of HCC by inhibiting E2F1-induced apoptosis [59]. As a downstream target of tumor suppressor p53, miR-34a functions as a link between p53 signaling and the cell cycle regulation by targeting cyclin D1, cyclin-dependent kinase 4 (CDK4) and CDK2 in HCC [60]. MiR-221 and miR-222 have been reported to target CDKN1B/p27/Kip1 and CDKN1C/p57/Kip2, while miR-223 participates in regulating the G2/M transition mediated by stathmin-1 [61]. In addition, miR-193b and miR-250b can suppress colony forming ability in vitro and tumorigenesis in vivo by inducing cyclin D1-mediated G1 phase arrest [62, 63]. 

A number of deregulated miRNAs are involved in the regulation of apoptosis. Bcl-2-modifying factor (Bmf) and p53 upregulated modulator of apoptosis (PUMA), two members of pro-apoptotic Bcl-2 family, are downregulated by miR-221 [64, 65]. The expression of three members of miR-106b-25 cluster, miR-25, miR-93, and miR-106b, is inversely correlated with Bim expression [59]. Conversely, the antiapoptotic members, B-cell lymphoma 2 (Bcl-2), induced myeloid leukemia cell differentiation protein (Mcl-1), and Bcl-2-like protein 2 (Bcl-w), are the targets of miR-125b [66, 67], miR-224 [68], miR-29 [69], miR-101 [70], and miR-122 [71]. Besides cell cycle regulation, miR-221 and miR-222 enhance the resistance to TRAIL-induced apoptosis by negatively regulating PTEN and metalloproteinase inhibitor 3 (TIMP3) [72]. Furthermore, let-7 miRNAs negatively regulate B-cell lymphoma-extra large (Bcl-xL) expression and enhance the sensitivity of HCC cells to apoptosis induced by Mcl-1- targeting anticancer drugs [73].

The activation of tyrosine kinase receptors (RTKs) initiates a signaling cascade that eventually leads to cell proliferation and survival. Many miRNAs have been shown to regulate the expression of proteins in RTK pathways. PTEN is downregulated by many miRNAs upregulated in HCC, such as miR-216a [74], miR-21 [75], miR-148a [76], miR-221/222 [72], miR-519d [77], and miR-29a [78], leading to the activation of PI3K/AKT/mTOR pathway. In addition, miR-7 regulates PI3K/Akt pathway by targeting to phosphoinositide 3-kinase (PIK3CD), mTOR, and p70S6K [79]. Restoring the expression of mTOR- and c-Met-targeting miR-199a-3p in HCC cells led to G1 arrest, reduced invasive capability, enhanced susceptibility to hypoxia, and increased sensitivity to doxorubicin-induced apoptosis [80]. c-Met can also be suppressed by other miRNAs including miR-23b [81], miR-1 [82], miR-198 [83], miR-449 [84], and miR-34a [85].

### 4.2. Regulation of Angiogenesis and Metastasis by Deregulated miRNAs in HCC

Angiogenesis and metastasis play important roles in the progression and recurrence of HCC. Aberrant angiogenesis and metastasis can be triggered by various stimuli from tumor microenvironment or/and intracellular signaling molecules that are subjected to the regulation of miRNAs. In an effort to identify potential miRNAs involved in the regulation of angiogenesis and metastasis, Santhekadur and colleagues unraveled a linear pathway in which staphylococcal-nuclease-domain-containing-protein-1 (SND1-) induced activation of NF-*κ*B resulted in miR-221 expression and subsequent induction of angiogenic factors angiogenin and chemokine (C-X-C motif) ligand 16 (CXCL16). Inhibition of either of these components resulted in significant inhibition of SND1-induced angiogenesis [86]. Hepatoma-derived growth factor (HDGF), a promoter of tumor angiogenesis, is a downstream target of miR-214 that is downregulated in HCC [87]. miR-122 can inhibit angiogenesis and intrahepatic metastasis by suppressing the expression of tumor necrosis factor-*α*-converting enzyme (TACE) [88]. In addition, downregulation of miR-29b and miR-125b in HCC contribute to the increased angiogenesis and metastasis through upregulating the expression of matrix metalloproteinase 2 (MMP2) and placenta-growth factor (PGF) [89, 90].

miRNAs are also involved in the metastasis through the regulation of epithelial to mesenchymal transition (EMT). For example, miR-10a promotes metastasis by regulating ephrin-type-A-receptor-4-(EphA4-) mediated EMT in HCC [89]. By downregulating Rho-associated coiled-coil containing protein kinase 2 (ROCK2) and histone-lysine N-methyltransferase (EZH2), miR-124 represses cytoskeleton reorganization and EMT, ultimately inhibiting the invasive and/or metastatic potential of HCC [173]. Meanwhile, p53 upregulates miR-200 and miR-192 family miRNAs to inhibit ZEB1/2-midated EMT [174]. More miRNAs involved in metastasis of HCC and their regulators and targets are listed in Table 1.

## 5. Clinical Potentials of miRNAs in HCC

### 5.1. miRNAs Related Genetic Variations and HCC Risk Prediction

Single nucleotide polymorphisms (SNPs) in some miRNAs or their targets are associated with risk of HCC (Table 2). Since the binding of a miRNA to its target mRNA is largely attributed to the seed sequence, even one nucleotide variation in the seed sequence would result in dramatic changes in the efficiency of miRNA-gene interaction. For instance, the rs11614913 (C→T) SNP in miR-196a-2 is positively associated with HCC susceptibility in Chinese [163, 164] and Turkish [165]. The “TTCA” insertion (rs3783553) disrupts the binding site in the 3′-UTR of IL-1alpha for miR-122 and miR-378, leading to the up-regulation of IL-1alpha expression and the promotion of HCC development [168]. However, conflicted results were achieved from three studies on the association between polymorphism of miR-499a and HCC risk in three populations with different ethnical backgrounds [159–161]. Therefore, polymorphisms of miRNAs or the 3′-UTR of their targets may be useful in HCC risk prediction.

### 5.2. miRNAs as Diagnostic and Prognostic Markers of HCC

The differential expression of miRNAs in hepatocellular carcinoma cells compared with their expression in normal hepatocytes indicates potential values of miRNA detection in HCC diagnosis and prognosis predication. For example, HCCs can be divided into three main clusters based on miRNA profiling [175]. Using a human miRNA microarray, Murakami and colleagues identified three significantly upregulated miRNAs and five downregulated ones in 25 HCC tissues compared with the nontumorous samples. The algorithm based on the detection of these deregulated miRNAs showed an overall prediction accuracy of 97.8% for HCC diagnosis. In addition, the expression levels of miR692, miR620, and miR618 were inversely correlated with the degree of HCC differentiation [176]. Downregulation of miR-139 was associated significantly with poor prognosis of patients and features of metastatic tumors including venous invasion, microsatellite formation, absence of tumor encapsulation, and reduced differentiation [107]. Inversely, high levels of miR-222 and C19MC miRNA were correlated with poor clinicopathological features such as increased risk of tumor recurrence and shorter overall survival [177, 178]. More studies on the clinical value of miRNAs detection in HCC are summarized in Table 3.

Extracellular miRNAs in the circulation are stable, suggesting that miRNAs may serve as novel diagnostic markers [191]. Yamamoto et al. first reported an increased amount of miR-500 in the sera of HCC patients and its levels dropped to normal after the surgical treatment [192]. Interestingly, Shigoka and coworkers found that the relative amount of miR-92a in the plasmas from HCC patients, was decreased compared with that from the healthy donors [193]. In addition, serum level of miR-25, miR-375, and let-7f can clearly separate HCC cases from non-HCC controls, and miR-375 level alone displays the potential for the detection of HCC [194]. So far, more than 20 circulating miRNAs have been identified as diagnostic markers of HCC.

### 5.3. Potential Roles of miRNAs in HCC Therapy

As miRNAs have confirmed to function as oncogenes or tumor suppressors and exogenous expression of tumor suppressor miRNAs or inhibition of onco-miRs resulted in alterations in malignance phenotypes of HCC cells in vitro, it might be possible to use artificial miRNAs to repress cancer development in vivo. Indeed, systemic administration of miR-26a, a miRNA expressed at high levels in normal tissues but downregulated in HCC, resulted in the inhibition of cancer cell proliferation, induction of tumor-specific apoptosis, and dramatic protection from disease progression without toxicity in a mouse model of HCC. These findings suggest that delivery of miRNAs that are highly expressed in normal tissues but lost in disease cells may provide a general strategy for miRNA replacement therapies without significant toxicity [58]. Two independent studies had reported that decreasing miR-221 level led to prolonged survival or a reduction of the number and size of tumor nodules in mice HCC models by using anti-miR-221 oligonucleotides or cholesterol-modified isoform of anti-miR-221 [195, 196]. On the other hand, restoration of miR-122 [88], miR-143 [132], and miR-124 [102] individually significantly inhibited tumorigenesis and metastasis in vivo.

Moreover, miRNAs have also been shown to influence sensitivity of tumors to anticancer drugs. HCC cells transfected with pre-miR-21 were resistant to the cytotoxicity induced by IFN-*α*/5-FU, and miR-21 expression in clinical HCC specimens was associated with the poor clinical response to the IFN-*α*/5-FU combination therapy [136]. In addition, miR-181b can enhance resistance of HCC cells to doxorubicin [134]. Therefore, antagomirs targeting miR-21 or miR-181b might be useful in increasing drug efficacy. In contrast, restoration of miR-122 in HCC cells renders them more sensitive to adriamycin and vincristine through downregulating the expression of multidrug resistance (MDR) proteins [197].

## 6. Conclusion and Perspectives

Deregulation of miRNAs significantly contributes to the development of HCC. miRNAs mainly functions to downregulate the expression of targeted genes. However, they may have other yet unknown functions including the activation of gene transcription. The discovery of new types or novel functions of miRNAs provides us with more and deeper insights into the molecular mechanism underlying the pathogenesis of HCC. On the other hand, the miRNA expression profiles altered in HCC paves the way to early detection and treatment of HCC. With the advantage of target multiple genes simultaneously, miRNAs as therapeutic targets would be more efficient than other single-gene targeted therapeutics such as RNAi-based therapy, thus representing a new avenue for the development of anti-HCC treatments.

## Figures and Tables

**Table 1:** MiRNAs deregulated in hepatocellular carcinoma (HCC).

miRNA	Expression in HCC	Regulator	Target	Involvement in cellular processes	References
Let-7a	Down	c-myc	Caspase-3	apoptosis; proliferation	[42, 91]
Let-7b	Down		HMGA2	apoptosis; proliferation	[92]
Let-7c	Down	EZH2; PPARalpha	Bcl-xL; c-myc	apoptosis; proliferation; cell growth	[43, 93, 94]
Let-7d	Down	c-myc		apoptosis; proliferation	[42]
Let-7f-1	Down	c-myc		apoptosis; proliferation	[42]
Let-7g	Down		Bcl-xL; COL1A2; c-Myc; p16(INK4A)	apoptosis; metastasis	[73, 95, 96]
miR-1	Down		ET-1;	proliferation	[97]
miR-101	Down	EZH2	Mcl-1; EZH2; EED; DNMT3A; SOX9	Colon formation; apoptosis; DNA methylation	[43, 70, 98, 99]
miR-122	Down	PPFP	Bcl-w; ADAM17; Wnt1	apoptosis; metastasis; Angiogenesis;	[71, 88, 100, 101]
miR-124	Down		PIK3CA	proliferation	[102]
miR-125a	Down		MMP11; VEGF-A; SIRT7	proliferation; metastasis; metabolism	[103, 104]
miR-125b	Down	EZH2; p53	LIN28B2; PIGF; Bcl-2; Mcl-1; Bcl-w; SUV39H1; SIRT7	proliferation; metastasis; angiogenesis; apoptosis; histone modification	[43, 66, 90, 104–106]
miR-139	Down	EZH2	ROCK2; c-Fos	metastasis	[43, 107, 108]
miR-138	Down		CCND3	Cell cycle	[109]
miR-145	Down		IRS1; IRS2; Oct4	Cell growth; tumorigenesis	[110, 111]
miR-195	Down		cyclin D1; CDK6; E2F3; LATS2	tumorigenesis; cell cycle; apoptosis	[57, 112]
miR-199a-3p	Down		mTOR; PAK4; caveolin-2	drug resistance; Cell growth;	[80, 113, 114]
miR-199a-5p	Down		DDR1; ATG7	invasion; autophagy	[115, 116]
miR-200a	Down	HDAC4	HDAC4	proliferation; metastasis;	[117]
miR-203	Down		Surviving	proliferation	[118]
miR-214	Down		*β*-catenin; HDGF	Cell growth; metastasis; angiogenesis	[87, 119, 120]
miR-219-5p	Down		GPC3	proliferation	[121]
miR-223	Down		STMN1	proliferation	[61]
miR-26a/b	Down	c-myc	CDK6; cyclin E1	Cell cycle	[122]
miR-29a	Down		SPARC	proliferation	[123]
miR-34a	Down	TGF-*β*	c-Met; CCL22	metastasis	[85, 124]
miR-375	Down		ATG7	Autophagy	[125]
miR-376a	Down		PIK3R1	apoptosis; proliferation	[126]
miR-449	Down		c-Met	proliferation; apoptosis	[84]
miR-450a	Down		DNMT3a	proliferation	[127]
miR-520b	Down		MEKK2; cyclin D1	Cell growth; proliferation	[63]
miR-7	Down		PIK3CD; mTOR; p70S6K	Tumorigenesis; metastasis	[79]
miR-10a	Up		EphA4; CADM1	EMT; metastasis	[128, 129]
miR-130a	Up		RUNX3	drug resistance	[130]
miR-135a	Up	FOXM1	MTSS1	metastasis	[131]
miR-143	Up	NF-*κ*B	FNDC3B	metastasis	[132]
miR-155	Up	NF-*κ*B	APC	proliferation; Tumorigenesis	[133]
miR-18a	Up		ERalpha	proliferation	[38]
miR-181b	Up	Smad4	TIMP3	Cell growth; tumorigenesis; metastasis	[134]
miR-182	Up		MTSS1	metastasis	[135]
miR-21	Up		PTEN; RHOB; PDCD4	metastasis; drug resistance	[75, 136–138]
miR-210	Up		VMP1; AIFM3	metastasis; apoptosis; proliferation	[139, 140]
miR-216a	Up	Androgen receptor	TSLC1	tumorigenesis	[141]
miR-221	Up	SND1	Bmf; CDKN1B/p27; CDKN1C/p57; DDIT4; Arnt	apoptosis; proliferation; Angiogenesis	[64, 86, 142–144]
miR-224	Up	HDAC1; HDAC3; EP300; NF*κ*B	RKIP; CDC42; CDH1; PAK2; BCL-2; MAPK1; API-5	Metastasis; proliferation; apoptosis	[68, 145–148]
miR-23a	Up	Stat3	PGC-1*α*; G6PC	Gluconeogenesis	[149]
miR-373	Up		PPP6C	Cell cycle	[150]
miR-301a	Up		Gax	metastasis	[151]
miR-490-3p	Up		ERGIC3	EMT	[152]
miR-519d	Up	p53	CDKN1A/p21; PTEN; AKT3; TIMP2	proliferation; invasion; apoptosis	[77]
MiR-550a	Up		CPEB4	Metastasis	[153]
miR-590-5p	Up		TGF-beta RII	Metastasis; proliferation	[154]
miR-615-5p	Up		IGF-II	Cell growth; migration	[155]
miRNA-657	Up		TLE1	proliferation	[156]

**Table 2:** SNPs in miRNAs and their target genes and the association with risk of HCC.

	Polymorphisms ID	Gene	Association with HCC Risk
Polymorphisms in miRNA genes	rs3859501	miR-371-373	Negative [157]
	rs7536540	miR-101-1	Positive [158]
	rs12375841	miR-101-2	Positive [158]
	rs2292832	miR-149c	Negative [159]
	rs3746444	miR-499a	No association [160] Negative [159] Positive [161]
	rs4938723	miR-34b/c	Positive [162]
	rs11614913	miR-196a-2	Positive [163–165] No association [159]
	rs2910164	miR-146a	No association [159, 166]
	rs999885	miR-106b-25 cluster	Positive [167]

Polymorphisms in miRNA target genes	rs3783553	IL-1alpha	Positive [168]
	rs16405	betaTrCP	Positive [169]
	rs17875871	IFNAR1	Positive [170]
	rs6147150	ErbB4	Positive [171]
	rs3917	COL1A2	Positive [172]

**Table 3:** Clinical relevance of deregulated microRNAs in HCC.

miRNA	Expression in HCC	Clinical relevance
miR-122 [179]	Down	Poor prognosis
miR-124 [173]	Down	More aggressive and/or poor prognosis
miR-139 [107]	Down	Poor prognosis
miR-145 [110]	Down	Shorter disease-free survival
miR-199b [180]	Down	Poorer overall survival, and progression-free survival rates
miR-22 [181]	Down	Poor survival
miR-26 [182]	Down	Shorter overall survival
miR-29 [69]	Down	Worse disease-free survival
miR-99a [183]	Down	Shorter survival
let-7g [95]	Down	Poor survival
miR-10b [129]	Up	Poor prognosis
miR-125b [184]	Up	Good survival
miR-135a [131]	Up	Poor prognosis
miR-17-5p [185]	Up	Worse Edmondson-Steiner grade, vein invasion, shortened overall survival and disease-free survival
serum miR-17-5p [186]	Up	Poor prognosis
miR-155 [187]	Up	Poorer recurrence-free survival and overall survival
miR-182 [135]	Up	Intrahepatic metastasis and poor prognosis
C19MC miRNA [177]	Up	Increased risk of tumor recurrence and shorter overall survival time
miR-21 [188]	Up	Poor prognosis
miR-221 [188]	Up	Poor prognosis
miR-222 [178]	Up	Shorter disease-free survival
20-miRNA signature [189]	Up/down	Metastases and recurrence
19-miRNA signature [190]	Down	Poor survival
